# Increasing Access to Mental Health Supports for 18- to 25-Year-Old Indigenous Youth With the JoyPop Mobile Mental Health App: Study Protocol for a Randomized Controlled Trial

**DOI:** 10.2196/64745

**Published:** 2025-01-30

**Authors:** Angela MacIsaac, Teagan Neufeld, Ishaq Malik, Elaine Toombs, Janine V Olthuis, Fred Schmidt, Crystal Dunning, Kristine Stasiuk, Tina Bobinski, Arto Ohinmaa, Sherry H Stewart, Amanda S Newton, Aislin R Mushquash

**Affiliations:** 1 Department of Psychology Lakehead University Thunder Bay, ON Canada; 2 Department of Psychology University of New Brunswick Fredericton, NB Canada; 3 Thunder Bay Counselling Centre Thunder Bay, ON Canada; 4 Dilico Anishinabek Family Care Fort William First Nation, ON Canada; 5 Ontario Native Women's Association Thunder Bay, ON Canada; 6 Department of Pediatrics University of Alberta Edmonton, AB Canada; 7 Department of Psychology, Neuroscience, and Behaviour Dalhousie University Halifax, NS Canada

**Keywords:** mental health, youth, Indigenous, First Nations, eHealth, mHealth, JoyPop, protocol, mobile mental health app, mobile app, Canada, mobile health, emotion regulation

## Abstract

**Background:**

Transitional-aged youth have a high burden of mental health difficulties in Canada, with Indigenous youth, in particular, experiencing additional circumstances that challenge their well-being. Mobile health (mHealth) approaches hold promise for supporting individuals in areas with less access to services such as Northern Ontario.

**Objective:**

The primary objective of this study is to evaluate the effectiveness of the JoyPop app in increasing emotion regulation skills for Indigenous transitional-aged youth (aged 18-25 years) on a waitlist for mental health services when compared with usual practice (UP). The secondary objectives are to (1) evaluate the impact of the app on general mental health symptoms and treatment readiness and (2) evaluate whether using the app is associated with a reduction in the use (and therefore cost) of other services while one is waiting for mental health services.

**Methods:**

The study is a pragmatic, parallel-arm randomized controlled superiority trial design spanning a 4-week period. All participants will receive UP, which involves waitlist monitoring practices at the study site, which includes regular check-in phone calls to obtain any updates regarding functioning. Participants will be allocated to the intervention (JoyPop+UP) or control (UP) condition in a 1:1 ratio using stratified block randomization. Participants will complete self-report measures of emotion regulation (primary outcome), mental health, treatment readiness, and service use during 3 assessments (baseline, second [after 2 weeks], and third [after 4 weeks]). Descriptive statistics pertaining to baseline variables and app usage will be reported. Linear mixed modeling will be used to analyze change in outcomes over time as a function of condition assignment, while a cost-consequence analysis will be used to evaluate the association between app use and service use.

**Results:**

Recruitment began September 1, 2023, and is ongoing. In total, 2 participants have completed the study.

**Conclusions:**

This study will assess whether the JoyPop app is effective for Indigenous transitional-aged youth on a waitlist for mental health services. Positive findings may support the integration of the app into mental health services as a waitlist management tool.

**Trial Registration:**

ClinicalTrials.gov NCT05991154; https://clinicaltrials.gov/study/NCT05991154

**International Registered Report Identifier (IRRID):**

DERR1-10.2196/64745

## Introduction

### Background

Transitional-aged youth refer to individuals aged roughly 15-26 years who are transitioning from childhood to adulthood, a time of developing independence and increased challenges [1]. In Canada, mental health difficulties among transitional-aged youth have increased across the past decade and during the COVID-19 pandemic [2,3]. Indigenous individuals within this age range experience a greater burden of mental health difficulties compared to their non-Indigenous peers [4,5], partly attributed to distal factors such as a family history of residential school attendance [6] and more proximal factors such as experiences of childhood adversity [7]. Stressors such as substance use, loss of culture, racism, socioeconomic status, and family instability also impact the well-being and mental health of Indigenous youth [8].

Mental health services are less accessible for youth living in remote and rural locations [9]. For instance, there is less use of outpatient-based mental health care and psychiatry services and higher rates of emergency department visits and hospital admissions for mental health-related reasons in Northern Ontario, a region of many different Indigenous communities [10]. In general, long wait times in Northern Ontario are similar to those experienced in other regions of the province [10]; however, wait times for counseling and therapy are particularly long in Thunder Bay compared to many other communities, with an average of 348 days [11]. Services that consider and incorporate culture are even more sparse. Long wait times for services not only delay care and contribute to prolonged distress and suffering but also affect interest and engagement in services once they are offered [12,13].

Mobile health (mHealth) innovations can increase access to mental health support. Specifically, smartphone apps can be an effective medium for improving mental health symptoms and quality of life [14,15] and for youth specifically [16]. Knowledge gaps exist, however, with respect to app effectiveness for those on a waitlist for services and for Indigenous youth. For instance, a recent review of waitlist interventions for youth did not include any app-based interventions [17]. Further, until recently, app evaluation studies seldom included Indigenous youth in the evaluations [18]. A review published in 2024 highlights several initial evaluations of apps with Indigenous youth that were focused on a variety of health-related outcomes, with a few of these focused on general mental health or coping and well-being [19]. Most of this research has been conducted with Indigenous populations outside of Canada, whose cultural values related to well-being may differ [20]. While there are apps geared toward Indigenous populations in Canada [21], they have not been evaluated among treatment-seeking youth, and some are primarily informational in nature or used for data collection only [22,23]. More research is needed to test the effectiveness of apps for addressing mental health among Indigenous youth [24].

In collaboration with an Indigenous-led agency informed by Anishinabek culture and located in Northern Ontario (Dilico Anishinabek Family Care; Dilico), in this study, the JoyPop app will be evaluated as a tool for supporting the mental health needs of Indigenous youth currently waiting for mental health services. The app was developed by researchers in collaboration with youth and service providers [25], and input from these stakeholders continues to inform its implementation and evaluation [26,27]. The app was designed to promote resilience to stress and adversity [25], targeting emotion regulation as part of this goal since it is linked to strengthening resilience [28]. Rather than targeting specific mental health diagnoses, focusing on emotion regulation is also in line with recommendations that apps target “transdiagnostic factors” to make the best use of resources [24], since difficulty with emotion regulation is common among different mental health concerns [29,30]. To accomplish this goal, app features include a mood rating feature to promote emotional awareness [31], breathing exercises to help with relaxation [32], relaxing sounds to help with sleep [33], a game and art feature helpful for distracting oneself from stressful situations [34,35], a journal focused on positive topics to promote well-being [36], and connection to one’s support network and professional helplines, if needed [37].

An initial evaluation of the JoyPop app showed that app use was associated with improvements in emotion regulation and depressive symptoms for transitional-aged youth starting university [38], who shared that they valued the opportunity to build awareness and regulation of emotions [39]. Another study conducted with an Indigenous community in Southern Ontario identified the potential utility of the app for youth in the community while also gathering knowledge on how cultural values were connected to the app features [26,27]. Prior to developing the current protocol, a pilot study was also conducted with Dilico and another local organization, in which youth identified benefits of using the app related to coping and mental health [40]. Service providers also expressed positive feelings about the app’s ease of use and how the app could be helpful for youth who are on the waitlist for services (eg, by increasing their comfort levels before services begin) [40].

In sum, it is important to identify beneficial transdiagnostic mental health supports for Indigenous transitional-aged youth in Northern Ontario. Considering past findings with the JoyPop app have been promising [26,27,38-40], a larger scale randomized controlled trial is warranted to more robustly evaluate its effectiveness. Providing this intervention while youth are on a waitlist for services may help reduce disengagement and the need to seek other services during this time.

### Objectives

The objectives of the study are in response to the goals and needs identified by the community partner, Dilico. Specifically, the primary objective is to determine the effectiveness of the JoyPop app compared to usual practice (UP) in improving emotion regulation among Indigenous transitional-aged youth (aged 18-25 years) who are awaiting mental health services. We hypothesize that youth receiving the app will show improvement in emotion regulation (small to medium effect) greater than that observed for youth receiving only UP.

The secondary objectives are to (1) compare change in mental health difficulties and treatment readiness between youth in each condition to better understand the app’s broader impact as a waitlist tool and (2) conduct an economic analysis to determine whether using the app while waiting for mental health services reduces other health service use and associated costs.

## Methods

### Overview of Study Design

This protocol was developed in collaboration with Dilico management and frontline staff to answer research questions of importance to the organization and the youth they serve. It was designed according to the Standard Protocol Items: Recommendations for Interventional Trials (SPIRIT) statement [41] (see Multimedia Appendix 1 for completed checklist). A pragmatic, parallel-arm randomized controlled superiority trial design will be used. Participants will be randomly assigned to the control (UP) or intervention (UP+JoyPop) condition in a 1:1 ratio using stratified block randomization. UP will involve waitlist monitoring practices at the study site, which include regular check-in phone calls to obtain any updates regarding functioning. In addition to UP, participants allocated to the intervention condition will receive the JoyPop app for 4 weeks and be asked to use it at least twice daily. Outcome measures will be administered to participants in both conditions during 3 assessments (first [baseline], second [after 2 weeks], and third [after 4 weeks]).

### Setting and Participants

Data collection will take place at Dilico Anishinabek Family Care, an Indigenous-led organization that provides culturally informed child welfare, mental health and addictions, and health services to Anishinabek children and adults. Programs and services are provided at individual, family, and community levels. This self-governed organization is situated in Fort William First Nation in Northern Ontario.

Youth will be informed about the study by waitlist case managers and with flyers and letters mailed and emailed to them. Youth will be eligible for trial participation if they are on the waitlist for mental health services at Dilico and between the ages of 18-25 years.

### Study Procedure

Table 1 describes participant progression through the study. A research assistant will contact interested youth by phone, text, or email to provide a brief description of the study. If a youth is interested and meets eligibility criteria, the research assistant will schedule their study orientation (as part of the baseline assessment). The research assistant will obtain informed consent during this orientation. As part of the informed consent process, youth will be made aware of local support services they can access should they experience distress during the study. They will also be made aware of the possibility that they are assigned to the control group so that they can make an informed decision about participation in light of this possibility.

**Table 1:** Participant timeline.

		–1	T1	T2	T3
Activity/assessment		Pre-study screening	Orientation/baseline assessment	2 Weeks/second assessment	4 Weeks/third assessment
Eligibility screen		✓			
Informed consent and condition allocation			✓		
**Intervention (UP+JoyPop; or UP only)**					
	Outcomes measured: emotion regulation, mental health, treatment readiness, and service usage		✓	✓	✓
	Outcome measured: app quality				✓—intervention group only
	Control group participants given access to app				✓

Following consent, participants will complete the baseline assessment measures. They will be encouraged to respond as honestly and accurately as possible and not how they think researchers might want them to. Any participant that endorses suicidal thoughts during the first assessment will see an onsite counselor who will conduct a risk assessment and intervene as needed. Following completion of the assessment measures, the research assistant will access the allocation envelope from the locked filing cabinet, open it to determine the allocation, inform the participant, and proceed with associated study tasks. Participants allocated to the intervention condition will be supported in accessing and learning about the app. All participants will return to Dilico after 2 and 4 weeks to complete the second and third assessments. Once they have finished the study at the end of their third assessment, participants in the control condition will learn about and receive access to the app.

To promote retention, participants will receive reminders about upcoming assessments via text and email. To incentivize completion [42,43], participants will receive cash compensation (CAD $20 [US $13.89] for baseline assessment; CAD $25 [US $17.36] for second assessment (after 2 weeks); CAD $30 [US $20.83] for third assessment (after 4 weeks); and an additional CAD $25 [US $17.36] if all assessments are completed). We will arrange transportation (taxis, bus fare) if required for participants. Promising retention rates were observed using these methods in a pilot study [44].

### Description of Intervention and Control Conditions

**Figure 1:**
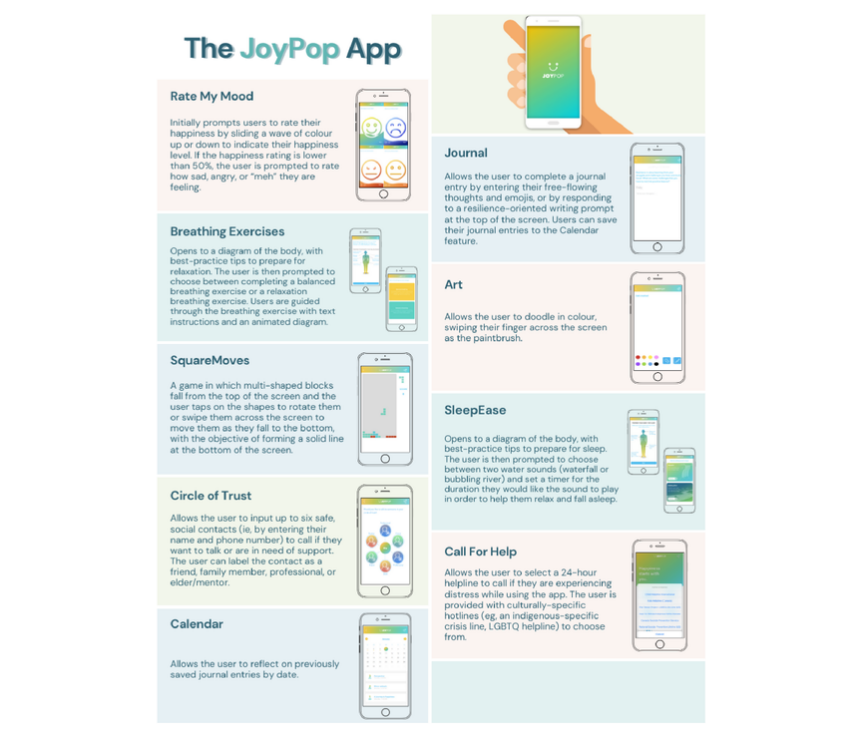
JoyPop features. LGBTQ: lesbian, gay, bisexual, transgender, and queer/questioning.

Participants in the intervention condition will be able to use the JoyPop app by downloading it onto their own device (if suitable). Participants who do not have an iPhone will be given a refurbished one containing only the app to use for the duration of the study. Participants will be asked to use the app at least twice daily for 4 weeks. The JoyPop app has features that allow participants to rate their mood, engage in relaxation exercises (ie, breathing), prepare for sleep, journal, play a Tetris-like game, create art, and reach out to their support system or suitable helplines. Figure 1 further describes these features. Internet connection is not required to use the app.

To promote engagement, youth will receive an email and SMS text message twice per day (at 8 am and 8 pm) reminding them to use the app. Outside of recommending using the app near the start and end of their day, we will not instruct or prescribe participants to engage with the app for any specific duration or pattern. Our intention is to evaluate the effects of using the app under real-world conditions in which users choose when and how often to use the app. Prior research suggests youth do use the app for the majority of the 4-week period [38,39]. Youth will also receive the usual waitlist management practice (UP).

Participants allocated to the control condition will only receive the UP. We chose this comparator to assess whether the JoyPop app could be a complementary support for individuals waiting for mental health services. This comparison creates the opportunity to test whether those who receive the app experience improved outcomes compared to those who do not receive it, representing “true” change after accounting for the regression to the mean phenomenon likely to affect both groups [45]. The use of a control condition addresses the limitations of our previous research in which all participants received the app [38].

### Randomization and Blinding

Stratified block randomization will be used to randomize participants to the control or intervention condition in a 1:1 ratio. An independent researcher outside of the research team will create the randomization sequence using a computer-generated sequencing tool [46] and then place allocations into numbered, opaque, sealed envelopes. These will be stored in a locked filing cabinet and only accessed by the research assistant who is with the participant.

To protect against bias, the principal and coinvestigators and the statistician conducting the analyses will be blinded to conditions. Trial participants cannot be blinded given the nature of this trial in which they are actively using the app or not. Research assistants will also not be blinded, as the condition assignment determines study procedures such as showing the participant how to use the app and providing them with the correct measures.

### Outcomes

The primary outcome is change in emotion regulation from the baseline assessment to the second assessment (after 2 weeks) and to the third assessment (after 4 weeks). Secondary outcomes are changes in mental health symptoms, readiness for treatment, and service usage from the baseline assessment to the second assessment and to the third assessment.

### Measures

#### Descriptive Measures

During the baseline assessment, participants will complete a demographics measure asking about their age, ethnicity, family composition, highest level of education, gender, sex at birth, sexual orientation, and living situation. Demographic variables that could change over time (ie, gender, sexual orientation, family composition, and living situation) will be reassessed at subsequent assessments. We will also measure symptom presentation during the baseline assessment using a modified version of the *DSM-5* (*Diagnostic and Statistical Manual of Mental Disorders* [Fifth Edition]) Self-Rated Level 1 Cross-Cutting Symptoms Measures (DSM-5-CCSM) [47] to characterize symptoms across 12 psychiatric domains (ie, depression, anger, irritability, mania, anxiety, somatic symptoms, inattention, suicidal ideation, psychosis, sleep disturbance, repetitive thoughts and behaviors, and substance use). One item pertaining to past suicide attempts was removed at the request of Dilico partners. Using the DSM-5-CCSM measure, respondents will report how much (or how often) they have been bothered by symptoms during the past 2 weeks. Items are rated on a 5-point scale (from 0=none at all to 4=severe or nearly every day) with the exception of items related to suicidal ideation and substance abuse, which are rated as either “Yes” or “No.” In a sample of adults receiving mental health services, most items in this measure except for the mania items have good test-retest reliability (intraclass correlation range=0.53-0.97) [48]. The measure also has good internal consistency (α=0.96) and criterion validity in terms of severity of impairment across life domains (*r*=0.84) in a community sample [49]. App usage data will be recorded in the JoyPop app with a timestamped record created each time the participant uses a feature.

#### Primary Outcome Measure

Emotion regulation will be measured with the Difficulties in Emotion Regulation Scale-Short Form (DERS-SF) [50,51], an 18-item self-report measure that asks respondents how often statements regarding their emotions have applied to them over the previous 2 weeks. Items are rated on a 5-point scale (ranging from 1=“almost never” to 5=“almost always”). Total scores range from 18 to 90, and subscale (strategy use, nonacceptance of emotion, impulsivity, ability to maintain focus on goals, awareness of emotions, and clarity of emotions) scores range from 3 to 15, with higher scores indicating greater difficulty regulating emotion. The DERS-SF demonstrates convergent validity via associations with symptoms of emotional disorders (eg, *r*=0.66 with the Beck Depression Inventory) [51,52]. In our pilot sample of Indigenous youth from Dilico, internal consistency for the total score ranged from α=0.87 to α=0.91 [53].

#### Secondary Outcome Measures

Mental health difficulties will be assessed with 2 measures: the Depression, Anxiety, and Stress Scale 21 (DASS-21) [54] and the Strengths and Difficulties Questionnaire (SDQ) [55,56]. The DASS-21 is a 21-item self-report measure that asks respondents how much specific statements regarding psychological distress applied to them over the past week. Items are rated on a 4-point scale (from 0=“never” to 3=“almost always”). Total scores range from 0 to 63, and subscale scores range from 0 to 21, with higher scores indicating greater distress. In clinical samples, this measure demonstrates convergent validity via correlations with other mental health measures (eg, *r*=0.85 between the Anxiety subscale and Beck Anxiety Inventory; *r*=–0.66 between the Depression subscale and Mental Health Questionnaire) [57,58]. In our pilot sample, internal consistency ranged from α=0.95 to α=0.96 across subscales. The SDQ is a 25-item self-report measure that asks respondents how true various statements have been for them. Items are rated on a 3-point scale (from 0=“not true” to 2=“certainly true”). Total scores range from 0 to 40, and subscale (emotional problems, conduct problems, hyperactivity, peer problems, and prosocial behavior) scores range from 0 to 10, with higher scores indicating greater difficulty (except for the prosocial scale for which the reverse applies). The SDQ has satisfactory internal consistency (α=0.80) and validity via its association with clinical diagnoses [56,59]. In our pilot sample, internal consistency was variable [53]; thus, we will ensure adequate psychometric performance in this study with a larger sample prior to analyses.

Treatment readiness will be assessed with the 4-item treatment readiness subscale of the Motivation for Youth’s Treatment Scale (MYTS) [60]. Items are rated on a 5-point scale (from 1=“strongly disagree” to 5=“strongly agree”), with the total subscale score calculated as the mean of item scores; higher scores indicate greater readiness. Groups with prior mental health service use and higher symptom severity tend to have higher average scores [60]. In our pilot sample, internal consistency ranged from α=0.80 to α=0.90.

Service usage will be assessed with 5 items generated by the research team that ask respondents how many times they accessed various health care services (ie, walk-in clinic, family doctor or nurse practitioner, emergency department, mental health counselor, and mental health hotline or phone support) over the previous 2 weeks.

### Statistical Analyses

#### Descriptives

We will describe the sample by calculating means and frequencies across demographic variables such as gender. We will also calculate mean scores on the DSM-5-CCSM to describe presenting mental health across 12 psychiatric domains. We will report internal consistency estimates and correlations between all measures to provide an indication of their reliability and convergent validity in the sample, given a lack of prior validation with Indigenous samples. With respect to app use among the intervention group, we will calculate the average frequency and duration of app use both in general and for each feature.

#### Primary Outcome

We will calculate the change in overall emotion regulation using the DERS-SF total score and the change in specific domains via the DERS-SF subscale scores. An independent statistician will use linear mixed modeling to test whether the change in emotion regulation is greater for those in the intervention condition relative to those in the control condition by including an interaction term between time and group [61]. We will evaluate the final sample to determine whether it is also feasible to analyze gender and baseline mental health symptoms as subgroup effects via interaction terms [62,63]. Analyses will be conducted with an intent-to-treat approach. Missing data will be handled with full information maximum likelihood estimation, which is recommended for avoiding bias in parameter estimates [64,65].

#### Secondary Outcomes

We will calculate change in mental health using total scores from the DASS-21 and SDQ, while we will calculate change in specific domains using the DASS-21 and SDQ subscale scores. We will calculate change in treatment readiness using the treatment readiness subscale from the MYTS as described. Similar to the primary outcome analyses, linear mixed modeling will be used to test whether the change in mental health symptoms and treatment readiness is greater for those in the intervention condition relative to those in the control condition by including an interaction term between time and group [61]. Covariates of gender and baseline mental health symptoms will again be included. With respect to service usage, we will estimate the costs of services used among participants in each condition and conduct a cost-consequence analysis to determine the incremental costs or savings associated with receiving the app [66].

#### Sample Size

We calculated the required sample size for a 2 (between subjects; treatment condition) by 3 (within subjects; time) mixed design, roughly estimating the statistical power needed for the planned linear mixed model for the primary outcome under the assumption of compound symmetry. We used parameters of *f*=0.2 (small to medium effect), =0.05, and power=0.95, resulting in a suggested sample size of 66 to achieve necessary power. We estimate 60% retention throughout the study (40% attrition) based on our initial research [53]; as such, an initial sample of 110 will be recruited.

### Ethical Considerations

#### Ethics Approval

The study protocol (version 1) was approved on December 16, 2022, by the Research Ethics Board at the Thunder Bay Regional Health Sciences Centre (file #100157). This Research Ethics Board acts as the Board of Record for clinical research projects led by Lakehead University researchers. The Research Advisory Committee at Dilico reviewed and approved all procedures. All participants will provide informed consent and will be made aware that they will receive counseling regardless of their choice to participate. Any required amendments would be reviewed by the Research Ethics Board, and participants would be informed by phone or email if such amendments directly impacted their experience in the study. We would also update the ClinicalTrials.gov registry (NCT05991154).

#### Mitigation of Harms

The risk of harm due to study participation is minimal. Participants may withdraw at any time by contacting the research team via phone or email. Any concerns or adverse events experienced or reported by participants to a research assistant would be reported immediately to the principal investigator (author ARM). The research team also meets weekly and will discuss any issues or concerns as they arise. All serious adverse events will be reported to the Thunder Bay Regional Health Sciences Centre Research Ethics Board using the Research Ethics Local Serious Adverse Event Reporting Form.

As described earlier, we will provide participants with contact information for local support services during the orientation, including walk-in counseling services and a local crisis line. Additionally, if recent suicidal thoughts are endorsed, an onsite counsellor will conduct a risk assessment with the participant and intervene as needed. As part of this risk assessment, the participant and counsellor will discuss whether continued study participation is recommended. Finally, as participants are on the waitlist for mental health services, they will be offered counseling services at some point following study completion (exact timing based on waitlist at the time).

#### Data Management and Confidentiality

Practices related to confidentiality follow the Canadian Tri-Council Policy Statement: Ethical Conduct for Research Involving Humans [44] and ethical guidelines at the Thunder Bay Regional Health Sciences Centre and Lakehead University. During recruitment, names and contact information of potential participants will be kept in a list only accessed by the research team. Once enrolled, the research assistant will assign each participant an ID number; both self-report and app data will contain only this ID number and no identifying information. The list connecting participant IDs to their names and contact information will be deleted upon study completion. The research assistant leading the orientation will inform participants about confidentiality and its limits during the informed consent procedure, including the procedure for notifying a staff member if recent suicidal ideation is endorsed; we will also provide this information in the information letter that is given to participants.

With respect to data management, a research assistant will enter responses from the hard-copy measures into a version of the measures hosted by Survey Monkey. Survey Monkey has SOC 2-accredited data centers [67] with physical security, including 24×7 monitoring, cameras, visitor logs, entry limitations, as well as dedicated cages for Survey Monkey hardware. Data in transit is encrypted using secure TLS cryptographic protocols. Usage data from the JoyPop app will be stored on a password-protected server within Canada and encrypted during transmission. In accordance with the Ownership, Control, Access, and Possession (OCAP) standards set by the First Nations Information Governance Centre [68], all data will be stored at Dilico; electronic measures and app data will be stored on a password-protected computer while hard-copy data will be stored in a locked filing cabinet. Data will not be shared with the public or outside third parties. Data will be retained for at least 7 years after study completion following Lakehead University policy.

#### Oversight and Monitoring

Author ARM, as the principal investigator, will oversee all trial activities and hold weekly meetings with research assistants to discuss the day-to-day running of the trial. Author ARM will consult with coinvestigators and meet on a quarterly basis to discuss the trial status and plan for future trial activities. Author ARM will also meet with collaborators from the local community partner (Dilico) to discuss ongoing implementation of the trial. Retention will be discussed on an ongoing basis with further strategies implemented if needed.

## Results

This research is funded through a Brain Canada—2021 Future Leaders in Canadian Brain Research grant, which began in October 2022. Recruitment began September 1, 2023, and is ongoing. As of July 2024, two participants have completed the study and attended all assessments. When the trial is complete, our community partner, Dilico, will review the findings via their research advisory committee, which is in line with OCAP principles [68]. Pending approval by the committee, results will be disseminated through academic conferences and publications as well as media interviews to reach the broader community. We will also share a summary of findings with participants who selected that they would like to receive this, as well as with staff at Dilico who are interested in the information. Pending positive results, avenues for future funding to support scalability will also be discussed with community partners.

## Discussion

### Overview

The current research will explore the impact of the JoyPop app on emotion regulation, mental health, and treatment readiness among Indigenous youth waiting for mental health services. Positive change among these variables and demonstrated cost-effectiveness associated with using the app would support adopting the app into routine waitlist management practices within the current partner organization. The degree and magnitude of findings within this clinical sample of Indigenous youth will be compared to prior work within a non-clinical sample in which use was associated with positive change in emotion regulation and depression symptoms [38]. We will also compare findings to another trial studying the effectiveness of the app among younger Indigenous youth [69] and another with non-Indigenous youth [70]. Such comparisons will allow identifying how to best use the JoyPop app as a supportive tool. Finally, positive findings would also be consistent with a growing emphasis on transdiagnostic approaches to mental health care. Specifically, findings may increase our knowledge about the link between emotion regulation and various mental health–related difficulties among Indigenous youth [29].

### Dissemination of Findings

When the trial is complete, our community partner, Dilico, will review the findings via their research advisory committee, which is in line with OCAP principles [68]. Pending approval by the committee, results will be disseminated through academic conferences and publications. We will also create infographics and videos to be shared broadly through team members’ networks, on social media, and through public presentations, which we will invite youth from the study to help develop and participate in. We will use media interviews to reach the broader community. We will share a summary of findings with participants who selected that they would like to receive this, as well as with staff at Dilico who are interested in the information. Pending positive results, we will discuss further scalability and potential integration of e-mental health solutions into services with the community partner, such as seeking funding to conduct a hybrid implementation/effectiveness study. We will also share findings with decision makers from other organizations who are similarly seeking solutions to meet youth mental health needs.

### Strengths and Limitations

Study strengths include the randomized controlled trial design, methods to promote retention and uptake of the intervention (eg, reminders and provision of phones and transportation), and evaluation of diverse outcomes, including both clinical outcomes and an analysis of service usage to understand the broader impact of the app within the health care system. The experimental design specifically improves upon the limitation of non-randomized earlier work [38]. An inherent study limitation is that participants are not blinded to condition assignment. A further limitation is that although the study was designed with few requirements surrounding app usage to allow for a more naturalistic examination, it is possible that in potential future nonresearch clinical settings, participants may engage with the app to a lesser extent when they are not actively involved in attending research sessions.

Given the nature of the intervention, an additional consideration is whether implementing an app-based intervention may contribute to problematic mobile device use. While this is an important consideration, the app does differ from social media and communication apps, which some research indicates are among those identified as the most “addictive” [71]. Future research, however, could evaluate whether accessing an app such as JoyPop encourages greater smartphone use overall. Future research could also incorporate scales assessing the risk of smartphone app addiction [eg, 72].

### Conclusions

Transitional-aged youth in Canada experience a heightened burden of mental health difficulties relative to adult populations, and Indigenous youth in this age range may be especially at risk [4,5]. Individuals who live in more remote or rural locations, such as in Northern Ontario, have less access to mental health care, which has negative impacts on well-being and engagement in future services [10,12]. This study, conducted in partnership with Dilico Anishinabek Family Care, will speak to whether adopting the JoyPop app as part of routine waitlist management practices may benefit Indigenous youth mental health and reduce additional service use during the waiting period. While an app is unlikely to remedy all mental health symptoms, it may be a useful tool for helping youth build skills and readiness in advance of formal counseling services.

## Appendix

Multimedia Appendix 1 SRIRIT (Standard Protocol Items: Recommendations for Intervention Trials) checklist.

## Abbreviations

DASS-21: Depression, Anxiety, and Stress Scale 21
DERS-SF: Difficulties in Emotion Regulation Scale-Short Form
Dilico: Dilico Anisinabek Family Care
DSM-5: Diagnostic and Statistical Manual of Mental Disorders (Fifth Edition)
DSM-5-CCSM: DSM-5 Self-Rated Level 1 Cross-Cutting Symptoms Measures
mHealth: mobile health
MYTS: Motivation for Youth’s Treatment Scale
OCAP: Ownership, Control, Access and Possession Standards
SPIRIT: Standard Protocol Items: Recommendations for Intervention Trials
SDQ: Strengths and Difficulties Questionnaire
UP: usual practice
