# Supplementary material for: Increasing Access to Mental Health Supports for 18- to 25-Year-Old Indigenous Youth With the JoyPop Mobile Mental Health App: Study Protocol for a Randomized Controlled Trial
Source: JMIR Res Protoc. 2025 Jan 30;14:e64745. doi: 10.2196/64745 (PMC11826949; doi:10.2196/64745)
Supplement: Multimedia Appendix 1 [file resprot_v14i1e64745_app1.pdf]

SPIRIT

STANDARD PROTOCOL ITEMS: RECOMMENDATIONS FOR INTERVENTIONAL TRIALS

SPIRIT 2013 Checklist: Recommended items to address in a clinical trial protocol and related documents*

Section/item	ItemNo	Description	Protocol
**Administrative Information**			
Title	1	Descriptive title identifying the study design, population, interventions, and, if applicable, trial acronym	Yes – see title
Trial registration	2a	Trial identifier and registry name. If not yet registered, name of intended registry	Under Abstract
	2b	All items from the World Health Organization Trial Registration Data Set	N/A
Protocol version	3	Date and version identifier	Under Ethics Approval
Funding	4	Sources and types of financial, material, and other support	Under Acknowledgements
Roles and responsibilities	5a	Names, affiliations, and roles of protocol contributors	Included
	5b	Name and contact information for the trial sponsor	Included
	5c	Role of study sponsor and funders, if any, in study design; collection, management, analysis, and interpretation of data; writing of the report; and the decision to submit the report for publication, including whether they will have ultimate authority over any of these activities	Funders have no role in the study design, data collection, analysis, or management, or reports/publication. Also reported under Conflicts of Interest.
	5d	Composition, roles, and responsibilities of the coordinating centre, steering committee, endpoint adjudication committee, data management team, and other individuals or groups overseeing the trial, if	Under Oversight and Monitoring

		applicable (see Item 21a for data monitoring committee)	
**Introduction**			
Background and rationale	6a	Description of research question and justification for undertaking the trial, including summary of relevant studies (published and unpublished) examining benefits and harms for each intervention	Under Introduction
	6b	Explanation for choice of comparators	Under Description of Intervention and Control Conditions
Objectives	7	Specific objectives or hypotheses	Under Objectives
Trial design	8	Description of trial design including type of trial (eg, parallel group, crossover, factorial, single group), allocation ratio, and framework (eg, superiority, equivalence, noninferiority, exploratory)	Under Overview of Study Design
**Methods: Participants, interventions, and outcomes**			
Study setting	9	Description of study settings (eg, community clinic, academic hospital) and list of countries where data will be collected. Reference to where list of study sites can be obtained	Under Setting and Participants
Eligibility criteria	10	Inclusion and exclusion criteria for participants. If applicable, eligibility criteria for study centres and individuals who will perform the interventions (eg, surgeons, psychotherapists)	Under Setting and Participants
Interventions	11a	Interventions for each group with sufficient detail to allow replication, including how and when they will be administered	Under Description of Intervention and Control Conditions
	11b	Criteria for discontinuing or modifying allocated interventions for a given trial participant (eg, drug dose change in response to harms, participant request, or improving/worsening disease)	N/A – There are no specified criteria for discontinuing or modifying the allocated intervention. Information regarding withdrawal and mitigation of harms is provided under Mitigation of Harms.

	11c	Strategies to improve adherence to intervention protocols, and any procedures for monitoring adherence (eg, drug tablet return, laboratory tests)	Under Description of Intervention and Control Conditions.
	11d	Relevant concomitant care and interventions that are permitted or prohibited during the trial	N/A - No concomitant care will be prohibited during study participation. Permitting access to concomitant care is an important feature of the study design, as we will directly measure utilization of other health services as an outcome variable in order to determine whether the JoyPop app is associated with a reduction in accessing such services.
Outcomes	12	Primary, secondary, and other outcomes, including the specific measurement variable (eg, systolic blood pressure), analysis metric (eg, change from baseline, final value, time to event), method of aggregation (eg, median, proportion), and time point for each outcome. Explanation of the clinical relevance of chosen efficacy and harm outcomes is strongly recommended	Under Measures and Statistical Analyses
Participant timeline	13	Time schedule of enrolment, interventions (including any run-ins and washouts), assessments, and visits for participants. A schematic diagram is highly recommended (see Figure)	Under Study Procedure, and depicted in Figure 1
Sample size	14	Estimated number of participants needed to achieve study objectives and how it was determined, including clinical and statistical assumptions supporting any sample size calculations	Under Sample Size
Recruitment	15	Strategies for achieving adequate participant enrolment to reach target sample size	Under Setting and Participants
**Methods: Assignment of interventions (for controlled trials)**			
Allocation:			

Sequence generation	16a	Method of generating the allocation sequence (eg, computer-generated random numbers), and list of any factors for stratification. To reduce predictability of a random sequence, details of any planned restriction (eg, blocking) should be provided in a separate document that is unavailable to those who enrol participants or assign interventions	Under Randomization and Blinding
Allocation concealment mechanism	16b	Mechanism of implementing the allocation sequence (eg, central telephone; sequentially numbered, opaque, sealed envelopes), describing any steps to conceal the sequence until interventions are assigned	Under Randomization and Blinding
Implementation	16c	Who will generate the allocation sequence, who will enrol participants, and who will assign participants to interventions	Under Randomization and Blinding
Blinding (masking)	17a	Who will be blinded after assignment to interventions (eg, trial participants, care providers, outcome assessors, data analysts), and how	Under Randomization and Blinding
	17b	If blinded, circumstances under which unblinding is permissible, and procedure for revealing a participant's allocated intervention during the trial	N/A - Participants and research assistants will not be blinded due to the nature of the intervention.
**Methods: Data collection, management, and analysis**			
Data collection methods	18a	Plans for assessment and collection of outcome, baseline, and other trial data, including any related processes to promote data quality (eg, duplicate measurements, training of assessors) and a description of study instruments (eg, questionnaires, laboratory tests) along with their reliability and validity, if known. Reference to where data collection forms can be found, if not in the protocol	Under Measures

	18b	Plans to promote participant retention and complete follow-up, including list of any outcome data to be collected for participants who discontinue or deviate from intervention protocols	Under Study Procedure
Data management	19	Plans for data entry, coding, security, and storage, including any related processes to promote data quality (eg, double data entry; range checks for data values). Reference to where details of data management procedures can be found, if not in the protocol	Under Data Management and Confidentiality
Statistical methods	20a	Statistical methods for analysing primary and secondary outcomes. Reference to where other details of the statistical analysis plan can be found, if not in the protocol	Under Statistical Analyses
	20b	Methods for any additional analyses (eg, subgroup and adjusted analyses)	Under Statistical Analyses
	20c	Definition of analysis population relating to protocol non-adherence (eg, as randomised analysis), and any statistical methods to handle missing data (eg, multiple imputation)	Under Statistical Analyses
**Methods: Monitoring**			
Data monitoring	21a	Composition of data monitoring committee (DMC); summary of its role and reporting structure; statement of whether it is independent from the sponsor and competing interests; and reference to where further details about its charter can be found, if not in the protocol. Alternatively, an explanation of why a DMC is not needed	N/A - A data monitoring committee was not established due to the minimal safety concerns associated with the trial. Concerns related to data collection will be reported by research assistants to the principal investigator on an ongoing basis and will be discussed at weekly research team meetings, as described under Mitigation of Harms.
	21b	Description of any interim analyses and stopping guidelines, including who will have access to these	N/A - There are no planned interim analyses or stopping guidelines.

		interim results and make the final decision to terminate the trial	
Harms	22	Plans for collecting, assessing, reporting, and managing solicited and spontaneously reported adverse events and other unintended effects of trial interventions or trial conduct	Under Mitigation of Harms.
Auditing	23	Frequency and procedures for auditing trial conduct, if any, and whether the process will be independent from investigators and the sponsor	N/A – There is no independent trial auditing process. Protocol adherence will be reviewed at weekly team meetings.
**Ethics and dissemination**			
Research ethics approval	24	Plans for seeking research ethics committee/institutional review board (REC/IRB) approval	Under Ethics Approval
Protocol amendments	25	Plans for communicating important protocol modifications (eg, changes to eligibility criteria, outcomes, analyses) to relevant parties (eg, investigators, REC/IRBs, trial participants, trial registries, journals, regulators)	Under Ethics Approval
Consent or assent	26a	Who will obtain informed consent or assent from potential trial participants or authorised surrogates, and how (see Item 32)	Under Study Procedure
	26b	Additional consent provisions for collection and use of participant data and biological specimens in ancillary studies, if applicable	N/A - Additional consent provisions are not required; biological samples will be obtained.
Confidentiality	27	How personal information about potential and enrolled participants will be collected, shared, and maintained in order to protect confidentiality before, during, and after the trial	Under Data Management and Confidentiality
Declaration of interests	28	Financial and other competing interests for principal investigators for the overall trial and each study site	Under Conflict of Interests
Access to data	29	Statement of who will have access to the final trial	Under Data Management and Confidentiality

		dataset, and disclosure of contractual agreements that limit such access for investigators	
Ancillary and post-trial care	30	Provisions, if any, for ancillary and post-trial care, and for compensation to those who suffer harm from trial participation	Under Mitigation of Harms
Dissemination policy	31a	Plans for investigators and sponsor to communicate trial results to participants, healthcare professionals, the public, and other relevant groups (eg, via publication, reporting in results databases, or other data sharing arrangements), including any publication restrictions	Under Results
	31b	Authorship eligibility guidelines and any intended use of professional writers	Under Acknowledgements
	31c	Plans, if any, for granting public access to the full protocol, participant-level dataset, and statistical code	N/A - The protocol is available on clinicaltrial.gov. Following OCAP™, participant-level data will remain in the possession of Dilico Anishinabek Family Care and will not be shared with the public or outside parties.
**Appendices**			
Informed consent materials	32	Model consent form and other related documentation given to participants and authorised surrogates	A copy of the information letter and consent form each participant will receive is included in Appendix 2.
Biological specimens	33	Plans for collection, laboratory evaluation, and storage of biological specimens for genetic or molecular analysis in the current trial and for future use in ancillary studies, if applicable	N/A. No biological specimens will be collected.

*It is strongly recommended that this checklist be read in conjunction with the SPIRIT 2013 Explanation & Elaboration for important clarification on the items. Amendments to the protocol should be tracked and dated. The SPIRIT checklist is copyrighted by the SPIRIT Group under the Creative Commons "Attribution-NonCommercial-NoDerivs 3.0 Unported" license
